# Pollutant-Induced Modulation in Conformation and β-Lactamase Activity of Human Serum Albumin

**DOI:** 10.1371/journal.pone.0038372

**Published:** 2012-06-07

**Authors:** Ejaz Ahmad, Gulam Rabbani, Nida Zaidi, Basir Ahmad, Rizwan Hasan Khan

**Affiliations:** Interdisciplinary Biotechnology Unit, Aligarh Muslim University, Aligarh, India; University of Hyderabad, India

## Abstract

Structural changes in human serum albumin (HSA) induced by the pollutants 1-naphthol, 2-naphthol and 8-quinolinol were analyzed by circular dichroism, fluorescence spectroscopy and dynamic light scattering. The alteration in protein conformational stability was determined by helical content induction (from 55 to 75%) upon protein-pollutant interactions. Domain plasticity is responsible for the temperature-mediated unfolding of HSA. These findings were compared to HSA-hydrolase activity. We found that though HSA is a monomeric protein, it shows heterotropic allostericity for β-lactamase activity in the presence of pollutants, which act as K- and V-type non-essential activators. Pollutants cause conformational changes and catalytic modifications of the protein (increase in β-lactamase activity from 100 to 200%). HSA-pollutant interactions mediate other protein-ligand interactions, such as HSA-nitrocefin. Therefore, this protein can exist in different conformations with different catalytic properties depending on activator binding. This is the first report to demonstrate the catalytic allostericity of HSA through a mechanistic approach. We also show a correlation with non-microbial drug resistance as HSA is capable of self-hydrolysis of β-lactam drugs, which is further potentiated by pollutants due to conformational changes in HSA.

## Introduction

Human serum albumin (HSA) is the most abundant multifunctional single chain protein in blood plasma. HSA plays important physiological and pharmacokinetical roles by binding and transporting exo- and endo-genous compounds [1], [2]. It also possesses some enolase, esterase and hydrolase activities [3]. Thus, this protein contains both binding and catalytic sites [4]. This is heart-shaped and 80×80×30 Å in size [5] and the molecular topology can be easily changed because of its flexible nature as demonstrated in physicochemical studies [6]. Transportation of solute is one of the best characterized roles of this protein which solublizes ligands and targets them to cells through binding to specific cell receptors. HSA bound to specific ligands are recognized by specific cellular receptors through the ligand-dependent conformations of this protein [7]. Additionally, upon ligand binding, albumin undergoes physiologically relevant structural changes as in case of HSA-oleate interaction. As a consequence of the alteration in the nature of the local environment surrounding Cys-34, the long chain of fatty acid regulates the radical-trapping antioxidant activity [8]. These ligand-dependent changes in protein conformations are specific to the type of ligands and more precisely to their capacity to accumulate in the binding pockets. The ligand-induced structural changes in HSA are suggested to mediate its role in receptor-mediated cellular interaction as well as solute transport in physiological conditions.

**Figure 1:**
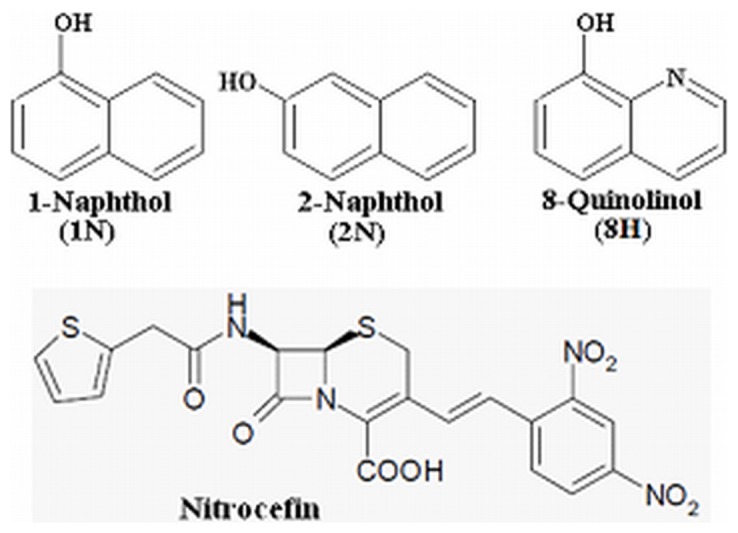
Structures. Pollutants (1N, 2N and 8H) and the substrate of HSA (nitrocefin).

**Figure 2:**
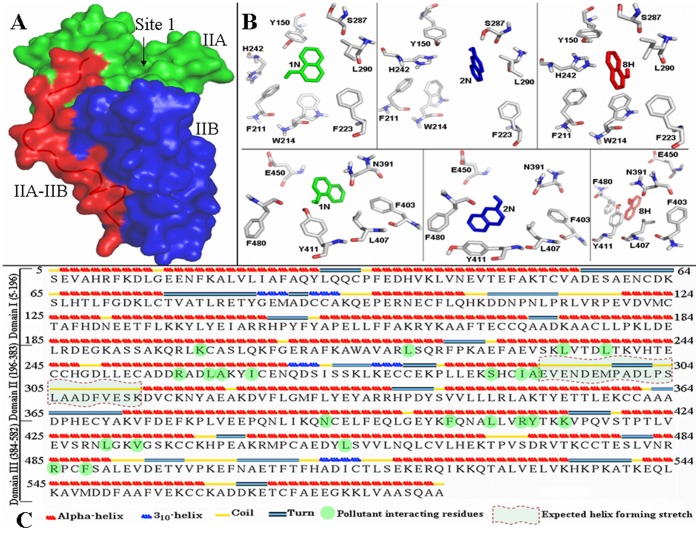
Pollutants interacting to the HSA. (A) The surface view of domain II of HSA (pdb:1AO6). The subdomain IIA (containing Sudlow binding site 1) is green, IIB is blue and IIA-IIB connecting region is red colored. The loop in ribbon view is clearly shown in background; (B) the flexible residues covering 1N (green), 2N (blue) and 8H (red) in site 1 (upper panel) and in site 2 (lower panel) are demonstrating π-π interaction of aromatic residues with these three pollutants; (C) the secondary structures in HSA sequence. The residues involved in HSA-pollutant interaction of Sudlow site 1 and 2 are highlighted in green. These figures are generated from our previous results [9].

We have studied the effect of pollutants on the structure and function of HSA. 1-naphthol (1N), 2-naphthol (2N) and 8-quinolinol (8H) shown in Figure 1 are direct or indirect (metabolite) organic pollutants and their accumulation in body can cause cyanosis, liver damage, nephritis, circulatory collapse and even death. A detailed study on the mode of interaction between HSA and these pollutants has been already performed and reported by our group [9] and the amino acid residues to which the pollutants bind are shown in Figure 2. In the present study, the effects of pollutant binding to HSA have been analyzed by a number of techniques. UV-visible, fluorescence spectroscopy, circular dichroism and dynamic light scattering are used to investigate the structural changes in protein associated with ligand binding. Here, our study offers not only direct proof for ligand-induced conformational alterations in protein, but also a clear understanding of the nature and after effects of these changes.

## Materials and Methods

### Materials

Fatty acid free human serum albumin (A1887), 1N (N2780) and 2N (185507) were from Sigma-Aldrich, USA, 8-quinolinol (8H), tris and hydrochloric acid were from Qualigens, India, whereas nitrocefin (484400) was a product of Calbiochem.

### Preparation of Solutions

All experiments were carried out in 20 mM tris-HCl buffer, pH 7.4. Fatty acid free HSA was used exactly as it was received. The protein concentration was spectrophotometrically determined (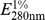 = 5.3) on Perkin–Elmer Lambda 25.

### Circular Dichroism

The isothermal wavelength scan and thermal denaturation studies of HSA in the absence and presence of pollutants were carried out with JASCO-J815 spectropolarimeter equipped with a Peltier-type temperature controller. The instrument was calibrated with d-10-camphorsulfonic acid. All the isothermal CD measurements were keeping at 37°C. Spectra were collected with 50 nm/min scan speed, 0.1 nm data pitch and a response time of 2 s. Each spectrum was the average of 2 scans. For the measurement of far-UV CD spectra (190–250 nm) the pathlength of cell was 0.1 cm while it was of 1 cm for near-UV CD (250–300 nm) spectra. The results were expressed as MRE (mean residue ellipticity) in deg.cm^2^.dmol^−1^, which is given by:
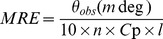
(1)where θ_obs_ is the observed ellipticity in degrees, *C*_p_ is the molar fraction and *l* is the length of the light path in centimeter [10]. All spectra were smoothed by the Savitzky–Golay method with 25 convolution width. The thermal denaturations were studied in the range of 25–90°C with 1°C min^−1^ temperature slope probed by far-UV CD at 222 nm and near-UV CD at 263 nm. To get the fractional populations of intermediates mediated elevated temperature, the CD values of unfolding at their respective temperatures were calculated by algebraic differentiation and the prominent peaks were considered as intermediates.

### Acrylamide Quenching Measurements by Steady State Fluorescence

Tryptophan fluorescence is used as a probe of local environment in a protein for determination of protein structure and dynamics. Intrinsic fluorescence studies were performed on Shimadzu 5301PC fluorescence spectrophotometer equipped with a constant temperature-holder with a 1 cm path length cell. The excitation and emission slits were set at 3 and 5 nm respectively. The concentration of protein was kept at 2 µM. Intrinsic fluorescence was measured by exciting at 295 nm to ensure selective excitation of Trp^214^ only and emission spectra was recorded in the range of 300–400 nm.

Acrylamide was used as a neutral dynamic fluorescence quencher due to the absence of electrostatic charge on this molecule. Aliquots of 1 M acrylamide stock solution were added to the HSA solution (2 µM) to achieve the desired range of quencher concentrations (0.01–0.2 M). As acrylamide absorbs highly at 280 nm, the excitation wavelength was set at 295 nm. The decrease value in fluorescence intensity at 330 nm was analyzed according to the Stern–Volmer equation:
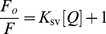
(2)where F_o_ and F are the fluorescence intensities in absence and presence of quencher [Q], acrylamide in molar concentration, *K*_sv_ is the Stern-Volmer quenching constant:
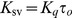
(3)where *K*_q_ is the bimolecular rate constant of the quenching reaction and τ_ο_ the average integral fluorescence life time of Trp_214_.

### Dynamic Light Scattering (DLS) Measurements

DLS measurements were carried out at 830 nm by using DynaPro-TC-04 dynamic light scattering equipment (Protein Solutions, Wyatt Technology, Santa Barbara, CA) equipped with a temperature-controlled microsampler. HSA (2 mg/ml) with the pollutants was overnight incubated at room temperature. The samples were spun at 10,000 rpm for 10 min and were filtered serially through 0.22 and 0.02 µm Whatman syringe filters directly into a 12 µl quartz cuvette. For each experiment, 20 measurements were taken. Mean hydrodynamic radius (*R*_h_) and polydispersity were analyzed using Dynamics 6.10.0.10 software at optimized resolution. The *R*_h_ was estimated on the basis of an autocorrelation analysis of scattered light intensity data based on translation diffusion coefficient by Stoke’s-Einstein relationship-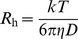
(4)Where *R*_h_ is the hydrodynamic radius, *k* is Boltzmann constant, *T* is temperature, *η* is the viscosity of water and *D* is diffusion coefficient [10].

### Enzyme Activation Kinetics

The alteration in hydrolase activity of HSA by pollutants (1N, 2N and 8H) was assessed individually by spectrophotometric method [11]. The assay was performed in 20 mM tris-HCl buffer, pH 7.4, using a β-lactam antibiotic, nitrocefin (a chromogenic cephalosporin) as the substrate by measuring its absorbance change at 514 nm. The substrate concentration ranged from 50 to 225 µM and the concentration of pollutants was 50 µM. The enzyme concentration was 10 µM. In another set of experiments, the pollutant concentrations ranged from 0 to 800 µM and the substrate concentration was 200 µM. Initial reaction time of 10 minutes was considered as pseudo first order kinetics so the slopes were measured after that duration by discarding the initial velocity data. The Michaelis-Menten equation was used to get the rectangular hyperbolic pattern of a typical enzyme-substrate reaction-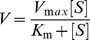
(5)where V and *V*_max_ is the initial and maximum velocity respectively, [S] is the substrate concentration, *K*_m_ is the Michaelis-Menten constant. The reciprocal of catalytic velocity was plotted against the reciprocal of substrate concentration at a constant activator concentration according to the equation (Lineweaver–Burk plot)-
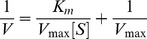
(6)


## Results

### Pollutant-induced Secondary Structural Changes

**Figure 3:**
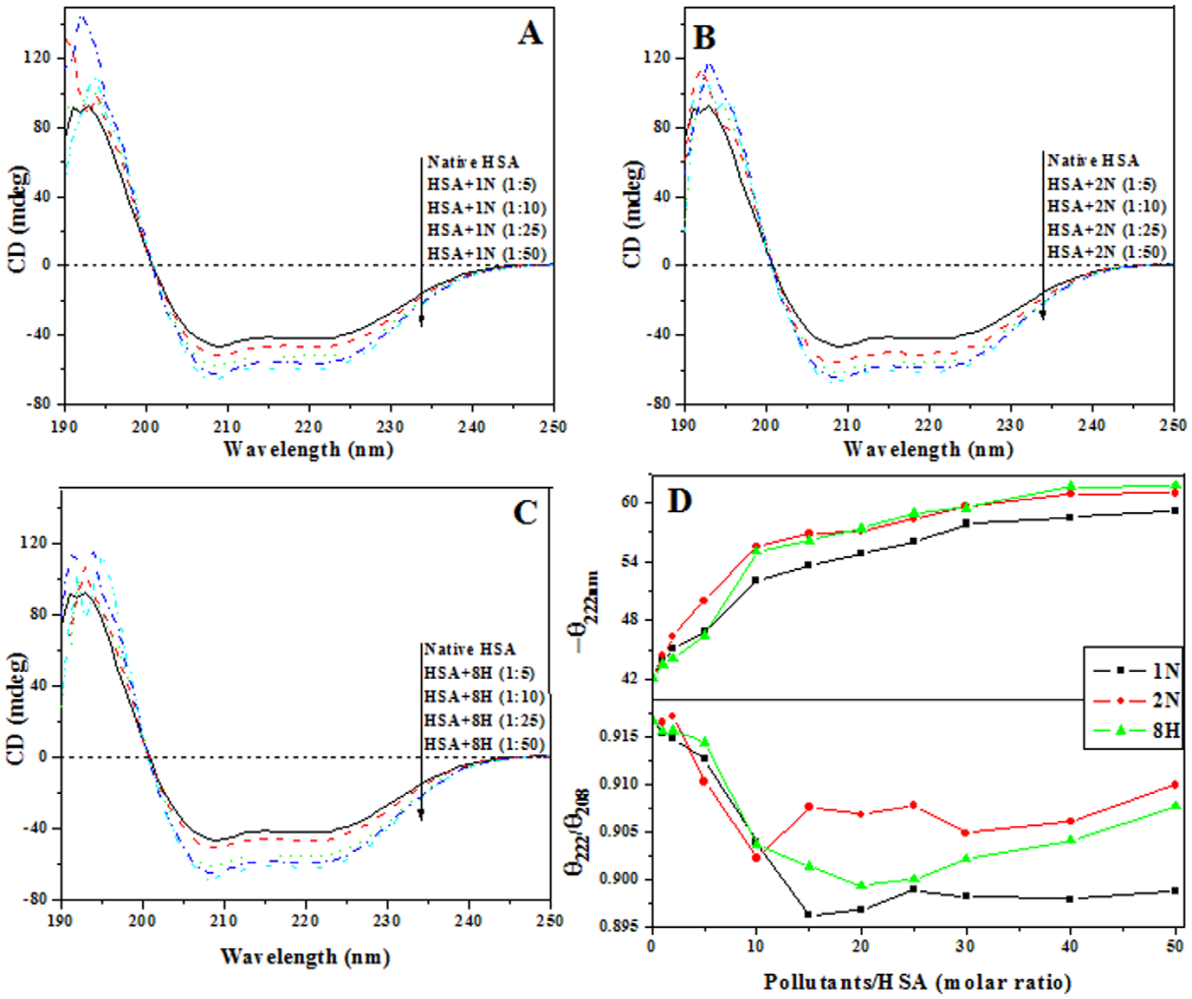
Secondary structural rearrangements. The far-UV CD spectra of HSA (5 µM) in 1N (A); 2N (B); 8H (C); while in (D) the upper panel corresponds to increase in helical contents whereas lower panel is for increase in 3_10_-helix upon increasing concentration of pollutants.

It is possible to estimate the secondary structures of a protein using far-UV CD spectra. A positive peak near 190 nm and two negative peaks near 208 and 222 nm are characteristic of a helical protein. From the spectra of Figure 3A to 3C, defined α-helical structures are detected for HSA in absence and presence of pollutants. As the pollutant concentration increased, a notable rearrangement of spectra occurred with increase in major minima due to intramolecular H-bonding rearrangement. These pollutant-induced alterations in secondary structures were quantified by K2D2, an algorithm-based neural network online software [12], [13]. The obtained results are summarized in Table 1. With the increase of pollutant concentrations, a significant increase in the helical structure is observed.

**Table 1:** Secondary structures of HSA in absence and presence of pollutants.

Conditions	α-helix (%)	β-sheet (%)	Random coil (%)
Native HSA	55.35	18.24	26.41
HSA+1N (1∶5)	58.29	19.19	22.52
HSA+1N (1∶50)	58.98	18.91	22.11
HSA+2N (1∶5)	57.91	18.10	24.99
HSA+2N (1∶50)	74.56	15.31	10.13
HSA+8H (1∶5)	76.62	15.12	8.26
HSA+8H (1∶50)	77.50	14.98	7.52

Spectra were scanned at different concentrations (pollutant/protein ratio in the range 0∶1 - 50∶1) and a single isodichroic point near 203 nm was obtained which is an indicator of two-state transition between a native protein and a greater helical protein. The shapes of all spectra were similar but this did not mean that the secondary structures in all cases were identical. As the pollutant concentration increases, the ratio of ellipticities at 222 and 208 nm decreases and afterwards becomes constant (Figure 3D). Lowering of [θ]_222 nm_/[θ]_208 nm_ values from unity is the sign of 3_10_-helix formation [14]–[16]. Hence, here the non-helical structures are expected to convert into 3_10_-helix.

### Pollutant-induced Tertiary Structural Changes

**Figure 4:**
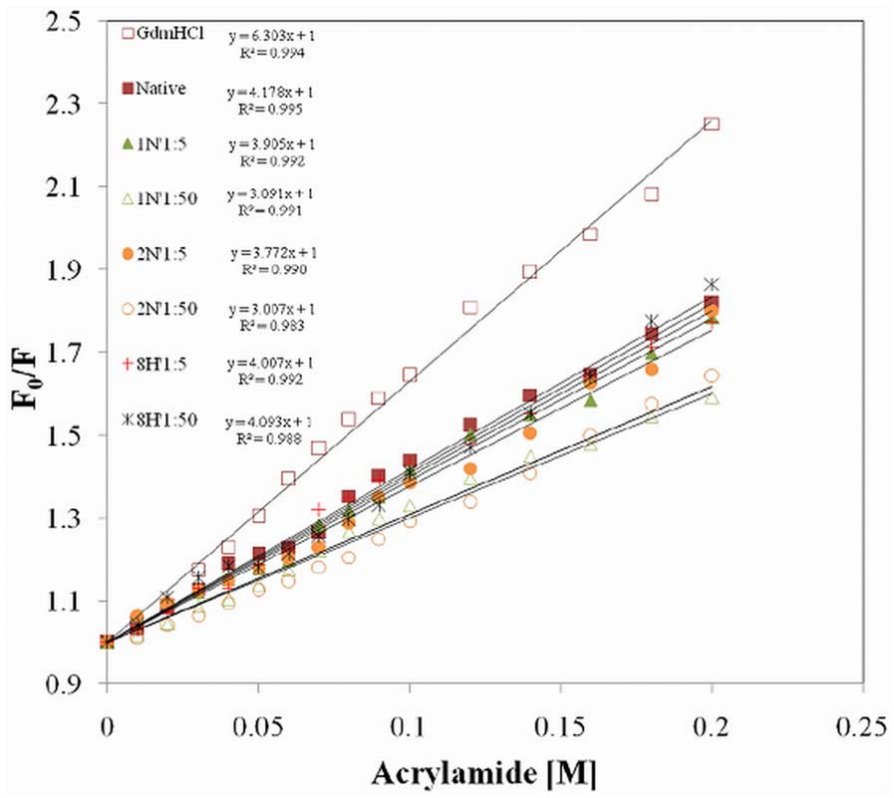
Stern-Volmer plot. Acrylamide quenching of native, denatured and pollutant complexed HSA (2 µM) at 37°C.

As HSA possesses a single Trp^214^ located on one side of the binding pocket in the second α-helix of subdomain IIA, it is feasible to study the accessibility to this moiety by a collisional fluorescence quencher, acrylamide, which can indicate the structural transitions induced by pollutants binding to albumin. Upon pollutant interaction with albumin, the extent of burial or exposure of Trp^214^ residue to the solvent was accordingly determined by monitoring the fluorescence emission intensity on increasing concentration of acrylamide as shown in the Figure 4. There were not any structural changes in albumin by acrylamide itself as the position of the emission peak was unaffected. The Stern-Volmer quenching constants of native and denatured albumin were taken as two extreme references (Table 2). From the obtained K_sv_ values, the values of *k*_Q_ were calculated by taking the average integral lifetime of HSA Trp^214^ fluorescence as 4.3 ns [17]. The values of *k*_Q_ were lower than the limiting diffusion constant K_diff_ of the biomolecules (2×10^10 ^M^−1^s^−1^) suggesting a dynamic quenching mechanism by the specific interaction of acrylamide with HSA. The straight line of the Stern-Volmer plot also implies that the quenching takes place on a simple collisional or dynamic basis in accordance with previous reports [18]. It was found that native HSA possess a smaller Stern-Volmer quenching constant (4.17 M^−1^) as well as lower accessibility of acrylamide to Trp^214^ than the denatured protein molecule (6.30 M^−1^) which indicates that Trp^214^ is located within the protein matrix. The addition of increasing concentrations of pollutants led to lower level of quenching even less than the native protein (Table 2). 1∶50 ([Protein]:[Ligand]) of 1N and 2N showed the maximum effect. Such changes are characteristic of a decrease in the polarity of Trp^214^environment. These results suggest that the structure of the protein matrix nearby Trp^214^ has become denser or more compact. The minimum amount of quenching was observed in the case of 8H in comparison to 1N and 2N. This was clear from the K_sv_ values, given in Table 2, which indicate that although these ligands did not result in decreased accessibility of Trp^214^ to the aqueous solvent, they did not denature the protein molecule.

**Table 2:** Acrylamide quenching results of HSA in absence and presence of pollutants.

Conditions	K_sv_ (M^−1^)	K_q_ (M^−1^s^−1^)	R^2^
Denatured HAS	6.30	1.46×10^9^	0.994
Native HSA	4.17	0.96×10^9^	0.995
HSA+1N (1∶5)	3.90	0.90×10^9^	0.992
HSA+1N (1∶50)	3.09	0.71×10^9^	0.991
HSA+2N (1∶5)	3.77	0.87×10^9^	0.990
HSA+2N (1∶50)	3.00	0.69×10^9^	0.983
HSA+8H (1∶5)	4.00	0.93×10^9^	0.992
HSA+8H (1∶50)	4.09	0.95×10^9^	0.988

### Pollutant-induced Alterations in Molecular Topology

**Figure 5:**
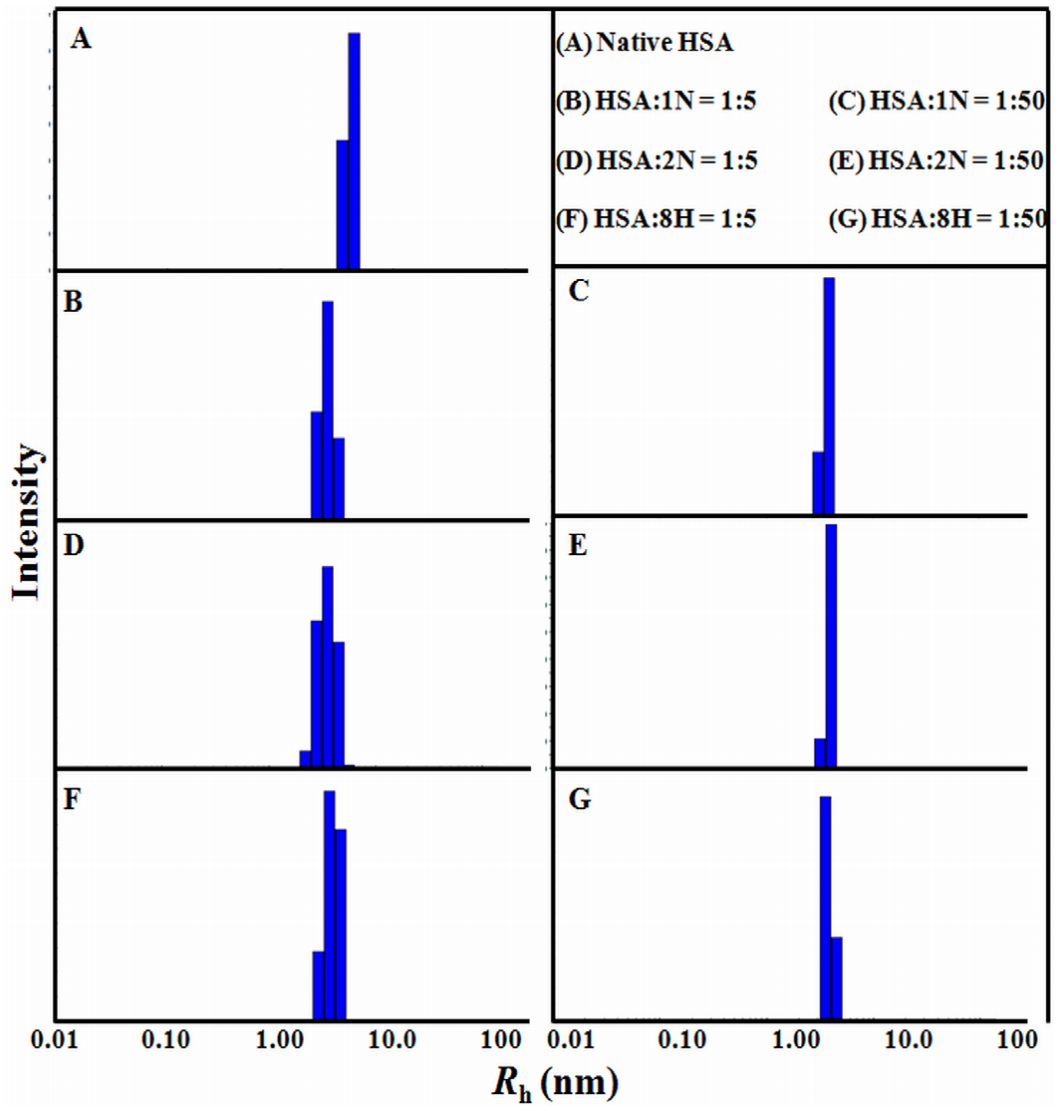
Dynamic light scattering of HSA-pollutant complex. Determination of hydrodynamic radii (*R*_h_) of HSA in absence and presence of pollutants.

It was clear from the above findings that upon interaction with pollutants, HSA undergoes conformational changes. The next step is to measure the size of such structural changes. Dynamic light scattering was employed to determine the hydrodynamic radii of native HSA and pollutant-complexed HSA. In Figure 5, the hydrodynamic radii (*R*_h_) of native HSA and HSA in presence of pollutants were calculated as in Table 3. The lower values of polydispersity (5.2–15.1) are indicative of homogenous species in the solution. The *R*_h_ value of 4.2 nm for native HSA is in agreement with previous observations [19]. The *R*_h_ values of HSA-complexed with pollutants were smaller than the native one. The reduction in hydrodynamic radii upon ligand binding may be due to the “collapsing” of protein around the ligand as it binds. This response may result in a decrease in the molecular volume due to a conformational change such as arabinose binding protein [20], maltose binding protein, calcium binding protein calmodulin and calmodulin-binding domain of calcineurin A [21], [22], D-galactose/D-glucose–binding protein, arginine kinase [23] which “close” on their ligand with a decrease in hydrodynamic volume upon ligand binding. This is also in accordance with another report where the *R*_h_ of TehB observed by DLS showed a 20% decrease upon tellurite and S-adenosyl-L-methionine binding [24]. The main reason for reduction in protein size is changes in the local dynamics of the protein, which are perturbed upon ligand interaction.

**Table 3:** Hydrodynamic radii and polydispersity of HSA in absence and presence of pollutants.

Conditions	*R*_h_ (nm)	Pd (%)
Native HSA	4.2±0.1	15.1±4.1
HSA+1N (1∶5)	3.6±0.2	6.3±1.9
HSA+1N (1∶50)	3.8±0.1	8.1±1.6
HSA+2N (1∶5)	3.7±0.1	5.2±1.1
HSA+2N (1∶50)	3.8±0.2	10.8±1.7
HSA+8H (1∶5)	3.8±0.1	10.2±1.4
HSA+8H (1∶50)	3.9±0.2	12.4±2.4

To ensure the full saturation of HSA binding sites, the molar concentrations of pollutants were taken as five times greater than that of HSA. Besides, 1∶50 was taken to analyze the solution effect on protein molecules imposed by higher concentrations of unbound pollutants. Here we saw that at extremely higher concentrations (1∶50), the hydrodynamic radii are slightly changed when they were compared with the radii of HSA in 1∶5 conditions. Consequently, it was suggested that the structural changes occurred only by molecules which are bound to the protein because of changes in secondary structures in the chain of protein molecules near the binding site.

### Thermal Stability of Albumin was Enhanced by Pollutants

**Figure 6:**
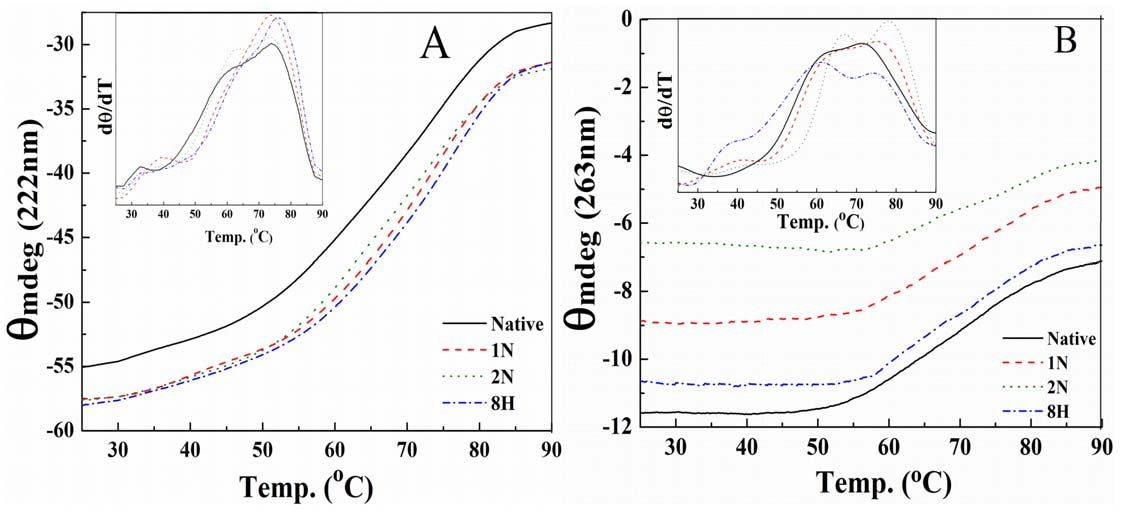
Thermal scan profiles of HSA in absence and presence of pollutants (1∶5) by CD. The values of (A) θ_222 nm_ for secondary structures and (B) θ_263 nm_ for tertiary structures were plotted against temperature. The insets are plots for the plus derivative of θ (mdeg) with respect to temperature (dθ/dT) versus temperature for the observation of fractional populations of domain specific intermediates.

HSA is a multidomain protein and its structure is stabilized by different inter-domain interactions. As each of the domains is an autonomous entity for its folding from nascent polypeptide, it is expected that its unfolding will also be independent of other domains. Hence, we have probed unfolding of HSA by means of both secondary and tertiary structures by far-UV CD (at θ_222 nm_) and near-UV CD (at θ_263 nm_) respectively. Figure 6A and 6B shows the curves of thermal unfolding transition for HSA in the absence and presence of pollutants. However, it may be possible to populate some intermediates between completely folded helical state and unfolded random state as the thermal unfolding curves are non-overlapping. This prominent non-overlapping of curves are also emphasizing about the fact that the disruption of secondary and tertiary structures in the protein are independent.

**Table 4:** Transition temperatures (T_m1_ and T_m 2_) and temperature mediated intermediates of HSA in absence and presence of pollutants (1∶5).

	2° Structure (θ_222 nm_)		3° Structure (θ_263 nm_)	
Conditions	T_m1_ (°C) for i_DII_	T_m2_ (°C) for i_DI_	T_m1_ (°C) for i_DII_	T_m2_ (°C) for i_DI_
**Native HSA**	61.0	73.3	62.0	71.3
(Fraction population)	(0.39)	(0.71)	(0.28)	(0.59)
**HSA+1N**	62.5	73.6	64.0	75.2
(Fraction population)	(0.35)	(0.67)	(0.18)	(0.67)
**HSA+2N**	63.0	73.7	66.8	78.1
(Fraction population)	(0.42)	(0.72)	(0.30)	(0.70)
**HSA+8H**	65.0	76.0	66.1	74.3
(Fraction population)	(0.40)	(0.71)	(0.36)	(0.65)

The denaturation curves show the pattern of Tm_1_ (domain II) and Tm_2_ (domain I) for secondary structural contents and tertiary contacts. In most of the cases (with and without pollutants), the helical content of domain II had broken up before the disruption of tertiary scaffold. Whereas, the same as above was not true for the denaturation pattern of domain I. The Tm_1_ and Tm_2_ values of pollutant-bound HSA are significantly higher than the native protein (Table 4). In case of native HSA, the major barriers to conformational transition are separation of all the three domains by loosening the interdomain contacts accomplished by hydrophobic/van der Waal/H-bonding/π-π interactions and this separation is followed by melting of individual domains into random coil unfolded states by weakening of intradomain interactions. Ligand-bound HSA has greater helicity or compactness compared to native albumin molecule which requires more energy to unfold. When a pollutant binds to HSA, the free energy contribution of ligand binding results in an increase in ΔG_U_, which frequently causes an increase in T_m_ and helix formation in random or unordered structures by packing through side chain-side chain interactions. The rigidity of the binding sites upon ligand binding simplifies the task of accounting for regional collapse or adjustment in the protein structure [25]. Denaturation of HSA at high temperature occurs by weakening of hydrophobic as well as polar interactions [6], which may also facilitate the pollutant-binding property of HSA. Here, enhancement in thermal stability is also implied by greater interaction of pollutants at high temperature which induce to greater helical formation in unordered protein segments.

**Figure 7:**
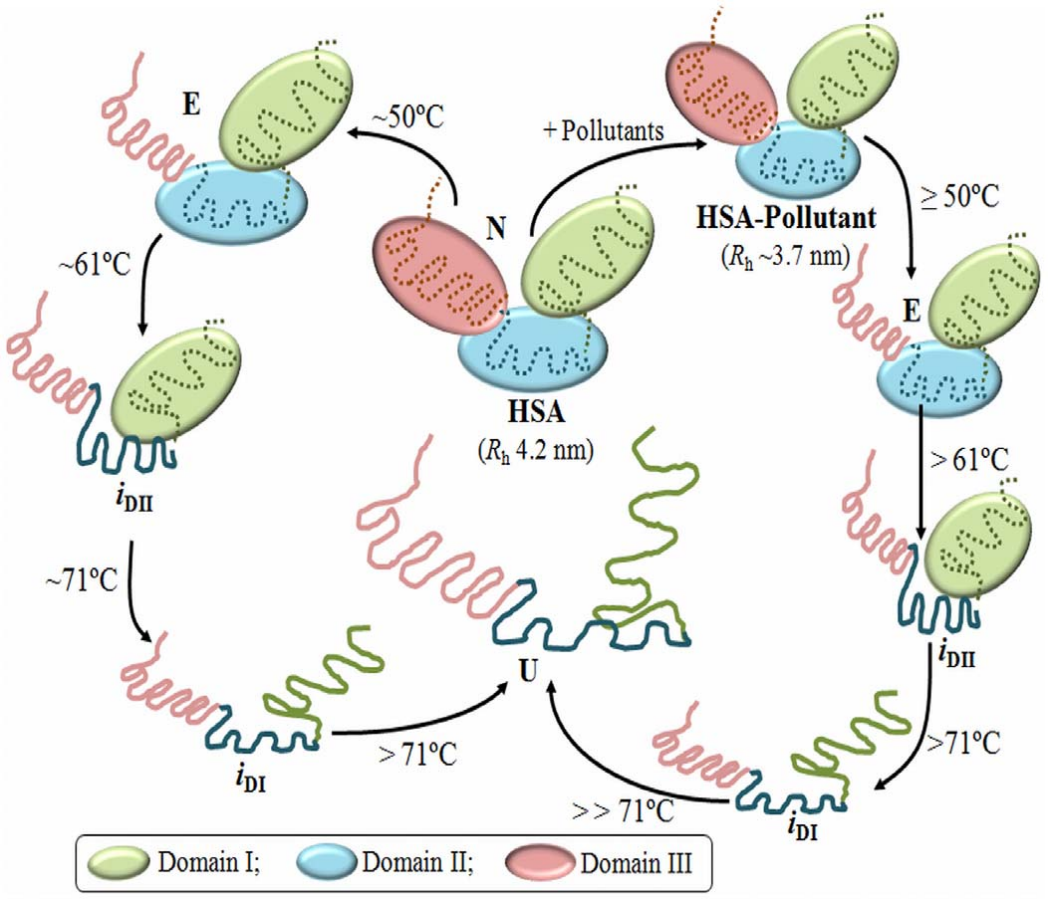
Difference in sequential unfolding of albumin (N) in absence and presence of pollutants. At mild temperature domain I and II separates and domain III melts out giving an expanded (E) conformation to the albumin. *i*_DII_ and *i*_DI_ corresponds to intermediates populated from the melting of domain II and I respectively and U is unfolded albumin.

The non-bonded interactions between planar aromatic rings are known as π-π interactions. These aromatic rings tend to form high-order clusters of parallel staggered axially symmetric system, which is a potential minimum configuration in the Lennard-Jones-Coulomb empirical potential calculation. The entropy driven π-stacking interactions stabilize the tertiary structure of protein where upon aromatic ring interaction with water molecules from the rings are released and the overall entropy of the thermodynamic system increases. Two aromatic boxes composed of Tyr^150^, Phe^211^, Phe^223^ and Trp^214^ in Sudlow site 1 and Phe^403^, Tyr411 and Phe^480^ in Sudlow site 2 are formed for the pollutants molecules (Figure 2B). The radial distances from the centered pollutants to each of the aromatic rings is between 3.5 and 4.0 Å, obtained from our previous study [9], fulfills the requirements of van der Waals and π–π interactions [26]. This radial distance is the shortest for 2N and this property of the ligand-binding site for 2N probably gives an unusual stability to the tertiary structures of the HSA molecule (Table 4). From the results of thermal denaturation, we found two populations of intermediates at two temperatures. The first temperature-induced intermediate (i_Dii_) was formed before the actual T_m_ of HSA and the other (i_Di_) was formed after the T_m_ values of albumin which is consistent with the previous reports on thermal denaturation of serum albumin where the first intermediate was considered the result of domain II disruption and the second of domain I disruption [27], meaning that thermal unfolding of HSA is a multistep and multistate process which is schematically summarized in Figure 7 and is consistent with the previous reports [27]. In the scheme, the smaller size of HSA complexed with pollutants than its native form as well as the delayed events of domain melting can be seen. Here, the difference in thermodynamic parameters obtained from secondary and tertiary structural probes revealed that thermal denaturation of HSA is not a monomolecular two state process in equilibrium. Taking into consideration equilibrium conditions simplifies our studies and allows us to compare the stability of HSA under the different conditions of various pollutants.

**Figure 8:**
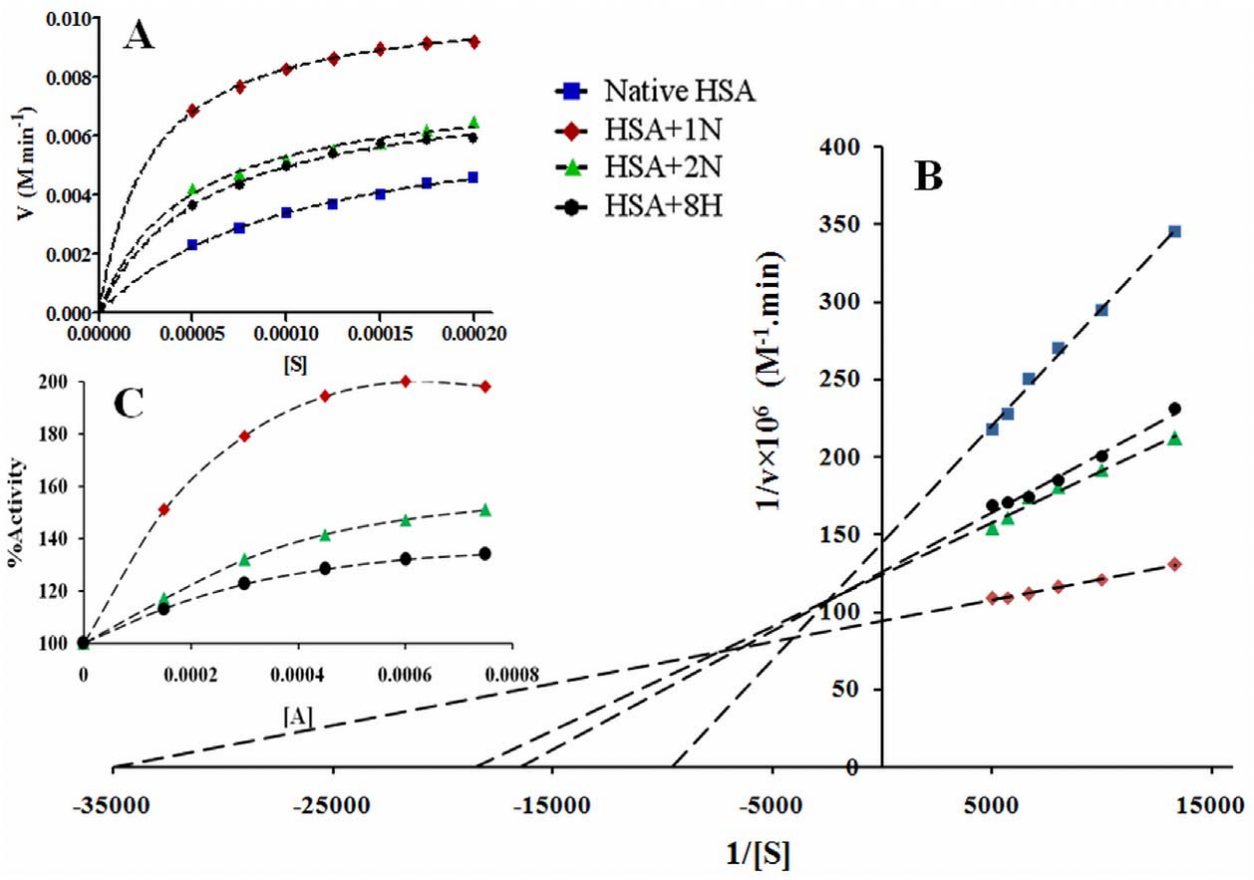
Enzyme kinetics for HSA. (A) is the Michaeil-Menton equation based; (B) is the Lineweaver-Burk plots of reaction velocity versus substrate concentration for enzyme kinetics of HSA in absence and presence of pollutants (1∶5); (C) is the plot of % activity against pollutant concentrations at a fixed substrate and HSA concentration.

In the presence of pollutants these intermediates were similar to native protein but increased temperature revealed the stabilization of not only domain II but also domain I. These results also revealed that domains I and II are unfolded in sequence and their opening is independent of one another [28], similar to loosening of the HSA structure in N↔F transition, which takes place primarily in domain III, without affecting domain I and II [29]. These populations were found in both conditions whether we were probing secondary structures or tertiary structures. The i_DI_ formed when all of the domains were completely melted but their fractional populations (0.59–0.72) are different from completely unfolded populations (1.0), hence, they were in partially unfolded states. This means there was some mutual collaboration among the domains in the unfolding process whereby some degree of structure was maintained even at high temperature. At elevated temperature the free -SH group of Cys^34^ possibly forms interdomain disulfide bonds [27].

### Activity of Albumin was Increased by Pollutants (‘Open’ to ‘Close’ Transition)

**Figure 9:**
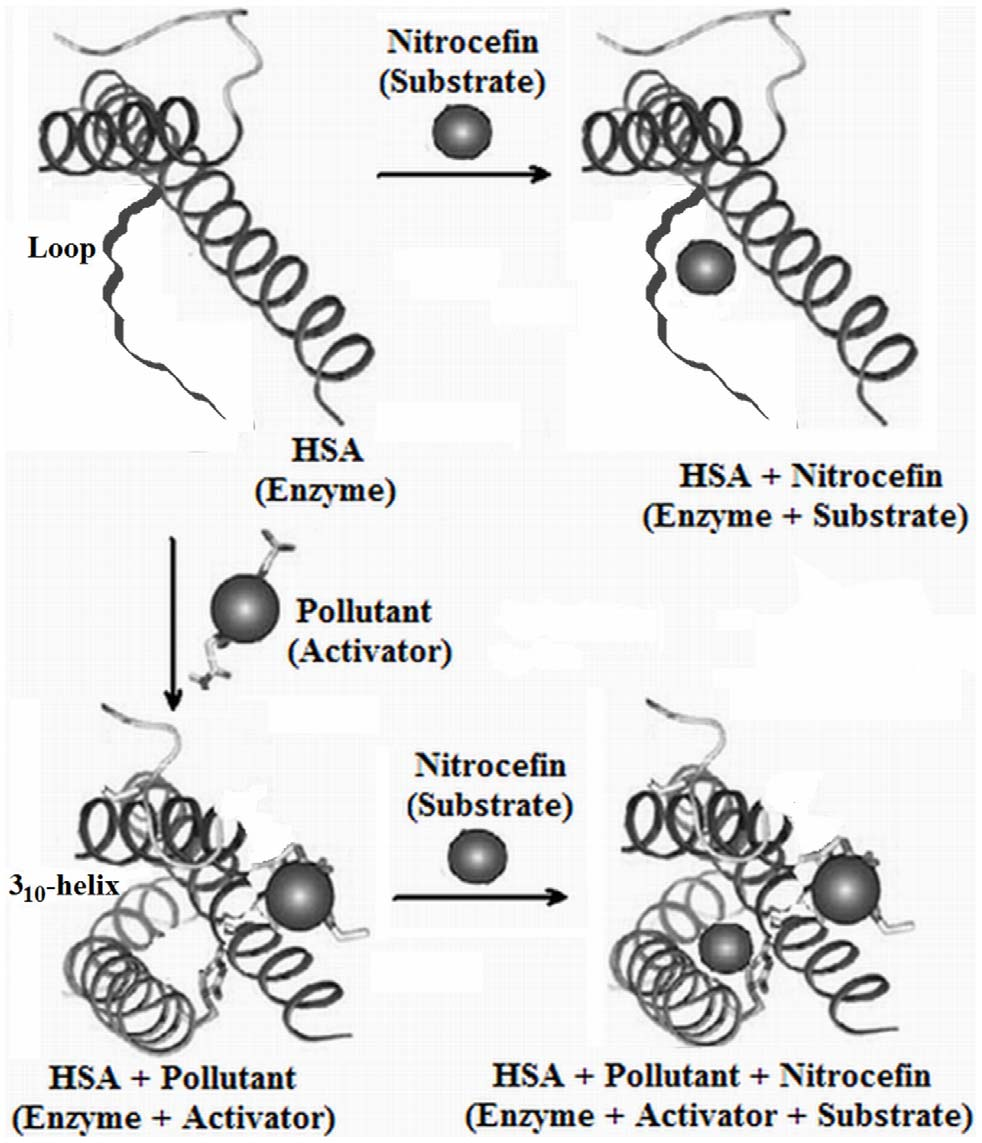
Schematic presentation. Nitrocefin (substrate) interacting with HSA (enzyme) in absence and presence of pollutants. The compactness of protein upon pollutant binding is clearly shown.

Figure 8A shows the pattern of nitrocefin hydrolysis by HSA in the absence and presence of a fixed concentration of these three pollutant molecules according to Michaelis-Menten equation. The rectangular hyperbolic curves are the characteristic feature of a true enzyme. Further, the reciprocal of substrate concentration against their respective reciprocal of degradation rate were plotted on the basis of Lineweaver-Burk equation (Figure 8B). The obtained values for all the extrinsic (V_max_) and intrinsic (*K*_m_, *k*_cat_) properties of an enzyme are listed in Table 5. In presence of pollutants the decrease in *K*_m_ from 1.05×10^−4 ^M to 2.86×10^−5 ^M reveals the increase in affinity and tighter substrate binding by the albumin. The turnover number (*k*_cat_) of the albumin complexed with pollutants is also increased up to ∼10 fold. The second order rate constant *k*_cat_/*K*_m_ indicates the catalytic efficiency and kinetic perfection of the enzyme in transforming substrates. The higher the *k*_cat_/*K*_m_ is, the better the enzyme works on that substrate. A comparison of *k*_cat_/*K*_m_ for the same enzyme with substrates in different conditions is widely used as a measure of enzyme effectiveness. The *k*_cat_/*K*_m_ value increased from 6.57×10^6^ to 3.71×10^7 ^M^−1^ min^-1^ in the absence and presence of pollutants (Table 5). The pollutant induced catalytic activation of HSA allows the reaction to approach the limit of maximum diffusion just like in an ideal enzyme (acetylcholinesterase) where every interaction with substrate yields a product and for these enzymes, from the diffusion theory, the value of *k*_cat_/*K*_m_ ranges 6×10^9^–6×10^10 ^M^−1^ min^−1^ [30]. The results of enzyme kinetics suggest that the pollutants act as activators. For the determination of activator category, whether it is essential or non-essential activation, the enzyme activity of HSA in presence of different concentrations of pollutant molecules is shown in Figure 8C and the obtained values are given in Table 5. Here, the non-linear enhancement in activity is observed. This shows a non-essential activation as the non-linearity is prerequisite of such type of activation. The mechanism of hydrolase activity of HSA occurs through irreversible coupling through acylation of nucleophilic group (−NH_2_, −OH, or −SH) of the protein with concurrent rupture of the β-lactam ring between the carbonyl carbon and nitrogen (Figure 1). At pH 7.4, the ε-amino group of lysine residues and guanidinium –NH_2_ group of arginine residues are positively charged. Hence these –NH_2_ groups are electrophilic but not nucleophilic, and cannot acylate the β-lactam ring. The –SH of Cys^34^ was unaffected because of the absence of any significant change in near-UV CD spectra of HSA-nitrocefin complex (data not shown) in the range between 250 nm to 255 nm. Hence the residues involved in enzymatic activity are –OH containing residues such as serine, threonine and tyrosine in the vicinity of the active site. A comparison of free and pollutant bound HSA reveals that a random coil of the protein molecule rotates by some angle, resulting in movements of the polypeptide chain and in closing the cleft where nitrocefin is bound (Figure 9). The above results point to a large conformational change consistent with the hinge motion of domains observed in other proteins. In the present study, the change in hydrodynamic radii upon ligand binding calculated from the DLS data is the same as that observed by other groups in different protein-ligand models from different technical approaches, validated by domain movements in solution. Hence, we can also say that pollutants induce domain movement in HSA.

**Table 5:** Enzyme kinetics parameters of HSA in absence and presence of pollutants (1∶5).

Conditions	Activity_max_(Fold)	*K*_m_(M)	V_max_(M min^−1^)	*k*_cat_(min^−1^)	*k*_cat/_ *K*_m_(M^−1^min^−1^)
HSA+1N	2.0	2.86×10^−5^	1.06×10^−2^	1.06×10^3^	3.71×10^7^
HSA+2N	1.5	5.41×10^−5^	8.10×10^−3^	8.10×10^2^	1.49×10^7^
HSA+8H	1.3	6.07×10^−5^	7.90×10^−3^	7.90×10^2^	1.31×10^7^
Native HSA	1.0	1.05×10^−4^	6.90×10^−3^	6.90×10^2^	6.57×10^6^
β-lactamase^a^	––	2.00×10^−5^	––	1.20×10^5^	6.00×10^9^
Acetylcholinesterase^b^	––	9.00×10^−5^	––	8.00×10^5^	1.07×10^10^
An ideal enzyme^c^	––	––	––	––	6×10^9^–6×10^10^

a The data of enzyme kinetics for a true microbial β-lactamase using benzylpenicillin as substrate;

b Acetylcholinesterase using acetylcholine as substrate is considered as an ideal enzyme;

c The theoretical values for an ideal enzyme.

## Discussion

HSA is a helical protein with a long extended loop links the two subdomains IIA and IIB. Helix formation is opposed by entropy of side chains [31], [32], driven by different types of forces such as backbone H-bonds, hydrophobic and van der Waals interactions. Factors that drive helix formation must originate in the backbone as the helix is an energetically favored structure [33]. These factors may be intrinsic to protein sequence in physiological conditions or they may be induced by external factors. The tendency of short unstructured peptide stretches in solution to form helical structures is induced by many cosolvents such as trifluoroethanol [34]. Even n-alcohols can achieve this effect but the molecular mechanism of helical stabilization is still not clear. It may be possible that amino acids having the propensity of helix non-formation in water are somehow changed to helix forming residues when this amino acid comes in the vicinity of pollutant molecules. Those factors may be side chain-side chain interactions and helix electric macrodipole-side chain interactions. The dipole moment of native HSA in water is 710 D [35], which would be altered in the presence of pollutant molecules. The pollutant molecules interact with amino acids in such a way that they orient the unordered peptide to have its macrodipole in the axial direction forming a folded regular helical conformation as phase separation of components with different polarity leads to huge dipole moments. Like water, these pollutant molecules are polar in nature, but have higher hydrogen bond accepting capability and lower hydrogen bond donating capability. The dielectric constant of water (80) is much higher than pollutants (1N, 4.03; 2N, 4.95) hence, they enhance the electrostatic effects. In the presence of these pollutant molecules, the free energy of loop regions increases and they tend to favor a conformation where amide groups of unstructured polypeptide backbone form helical structure by hydrogen bonding.

From all of our experiments dealing with the secondary and tertiary structure of HSA in the presence of pollutant molecules, it becomes apparent that upon interaction between HSA and pollutants, the affinity of the protein for its substrate nitrocefin is enhanced. This is similar to a previous report where the binding of a ligand in binding site 1 is enhanced three times by the other ligands which bind elsewhere but in close proximity to the binding site 1 [36], [37]. Oleic acid and ibuprofen inhibit the β-lactamase activity of HSA [11]. There are two common sites for these inhibitors: Sudlow’s site 2 (domain IIIB) and interconnecting region of domain IIA-IIB. Pollutants also bind in site 2 of subdomain IIA as observed in our previous study [9]. The activity enhancement in the presence of pollutant means nitrocefin and pollutant cannot bind at the same site. According to [38], if oleic acid does not bind at Sudlow site 2 (ibuprofen binding site), then how it will act a competitive inhibitor for site 2 nitrocefin hydrolysis. The maximum nitrocefin hydrolysis rate of HSA is achieved above 50°C [39] whereas the domain III melts below 50°C [40] and this means nitrocefin does not bind in domain III. Hence, nitrocefin binds in the IIA-IIB domain connecting region where both oleic acid and ibuprofen can bind. Pollutant binding to HSA induces a significant structural alteration in HSA because the pollutant bind to binding site 1, adjacent to the interface between subdomains IIA and IIB (Figure 2A), which lies close to the nitrocefin binding site. In the absence of pollutant molecules, the unstructured linker connecting the helices *h*6 of IIA and *h*1 of IIB possess a wide opening gorge, which can’t fulfill the requirement for proper alignment of catalytic functional groups on the protein. However, pollutants occupying site 1 induce helix formation in this loop which pulls the side chains of nearby interacting residues and causes them to come together in an active site near the substrate, altering the environment for proper non-covalent interactions to overcome the physical and thermodynamic reaction barriers. In other words, the interaction of these naphthols to HSA generates a different orientation of the active site residues where the non-covalent interactions are coordinated in such a way that the active site binds the transition state more tightly by giving a better fit for the substrate resulting in lowering of the free energy for substrate hydrolysis.

The advanced glycation end-product (AGE) or scavenger receptor recognizes only glycated albumins. Similarly, cellular receptors identify and preferentially bind albumins bound to specific ligands. Another possible recognition mechanism may be a ligand-dependent conformational change in the HSA molecule. Hence, we provide insight into the molecular basis of HSA physiology by characterization of ligand-dependent conformational changes. The ability of a protein to recognize its substrate is controlled by other small molecules. These accessory molecules have either the capability of binding with protein or they change the intrinsic property of protein by changing the environment. In both cases the protein structure is altered, which facilitates the interaction between protein and substrate. Hence, these molecules provide a stimulus that converts an extended coil to a collapsed globule upon interaction with the protein in the vicinity of active site for substrate. β-lactam antibiotics are usually co-administered with a suitable microbial β-lactamase inhibitor but consideration must be taken to prevent the self-hydrolysis of these antibiotics by drug carriers which can reduce the bioavailability of this drug. The biggest concern will be when the patient is exposed to naphthols and naphthol-derived pollutants which act as non-essential hetertropic allosteric activators. The present study provides helpful insight when administering drugs because enzyme activity can be altered and allosteric sites can be drug targets. β-lactamase activity of HSA is altered upon the formation of albumin-pollutant complex due to conformational change. This means that naphthols may act as regulators of enzymatic activity of albumins. Hence, regulation of ligand-binding is of paramount importance in biochemistry. Pollutant-saturated albumin molecules show higher β-lactamase enzyme activity, which is likely due to interactions between two distinct pollutants and β-lactam binding sites. These interactions provide a conformational change, which distorts the orientation of β-lactam binding site. This may also be due to change in secondary structure of the protein upon binding with pollutants by changing the hydrogen bonding pattern of the backbone as well as the side chain. As life is granted only through binding events which amend environment and signaling pathways, the ligand-induced structural changes in protein should be a matter of wide-ranging studies. We summarize that there is pollutant-induced helix increment in HSA which is composed of α-helix, 3_10_-helix, turn and loop. Though the turns mostly posses proline which form a kink and in aqueous solutions, they are a helix breaker. The helical propensity of proline was found to be greatly enhanced in the organic solvents [41]. This means the turns and loops in HSA can be converted into helices. As the ratio of [θ]_222 nm_/[θ]_208 nm_ continuously decreases, the newly formed helices are of the 3_10_-helix type. All three pollutants bind in site 1 (domain II) and site 2 (domain III) but the affinity and strength of binding in site 1 is stronger. The nitrocefin binding site exists in domain II and site 2 is far apart from the nitrocefin site. This means only non-helical portions around or in domain II are responsible for conversion to helical one. The transition from lower to higher helical conformation is followed by closer packing of the molecule; hence, there is a decrease in size and higher thermal stability of protein molecule. Therefore, geometric fitting by helical packing occurs due to the binding of pollutants. The active site for nitrocefin (a β-lactam antibiotic) is composed of a long non-helical region in domain IIA-IIB adjacent to the site 1 of domain IIA which is helical in nature. Most of active sites of enzymes are situated close to a helix in such a manner that the dipole electrical field may increase reaction rates. The β-lactamase activity of HSA increases in the presence of pollutants, which work as allosteric modulators to increase the enzyme-substrate reaction. This is because the interactions of the pollutants influence the nearby active site to adopt a different conformation which can work better than in its native conformation. To measure the HSA concentration, the enzymatic activity of HSA has been assessed [42].

Conclusively, it has already been shown that the enzymatic properties of HSA ligand binding are stereospecific [43]. Our study adds that enhanced enzymatic activity by activators is not only stereospecific (1N, 2N and 8H) but also stereoselective as the hydrolase activity of HSA for β-lactam is different in presence of position isomers of naphthols (1N and 2N). From the present study we can postulate that *in individuals who are exposed to these pollutants-* 1: the determination of HSA concentration by enzymatic assay will give a false reading; 2: the resistance towards β-lactam antibiotics will be more prominent; 3: the dose regime of β-lactam antibiotics and other binding site 2 specific drugs should be carefully considered. Our study reveals that ligand-induced protein stabilization can improve the quality of most of proteins and also assist crystallization procedures for an unordered protein or protein segment. Here the mechanism of achieved activity by a natively unfolded protein upon binding with their desired ligand for a function can also be deduced. Therefore, our study also suggests a mechanism for the conformational change in unfolded proteins upon binding with the desired ligand. A detailed understanding of biochemical and biophysical processes involved in HSA-catalyzed reactions provides a critical tool for new drug discovery, for the large-scale industrial synthesis of chemicals, and for better appreciation of the chemistry of allosteric activation.
